# Differential monocyte microrna expression profiles in children with active systemic juvenile idiopathic arthritis

**DOI:** 10.1186/1546-0096-12-S1-P59

**Published:** 2014-09-17

**Authors:** Grant Schulert, Ndate Fall, Nan Shen, Alexei Grom

**Affiliations:** 1 Pediatrics, Cincinnati Children's Hospital Medical Center, Cincinnati, USA

## Introduction

Systemic juvenile idiopathic arthritis (SJIA) is an autoinflammatory disease of childhood, and the predominant effector cells are mononuclear phagocytes rather than lymphocytes as in autoimmune diseases such as RA. Previous gene expression data has shown that monocytes in SJIA have a novel phenotype with clear proinflammatory activation as well as features of alternative activation. This aberrant phenotype may contribute to the potential of these children to develop macrophage activation syndrome (MAS). What controls monocyte/macrophage differentiation in SJIA is unknown. MicroRNA are small, non-coding RNA that serve as transcriptional negative regulators to fine-tune gene expression programs involved in cell differentiation, metabolism and immunity. There is growing evidence that miRNA contribute to the pathogenesis of human disease, including adult rheumatoid arthritis (RA). These regulators have also been implicated in controlling differentiation of monocytes and macrophages. However, miRNA expression in SJIA has not been examined.

## Objectives

Here, we examine miRNA expression profiles in peripheral blood monocytes from children with SJIA.

## Methods

We enrolled children with active SJIA, defined as presence of active arthritis or systemic features, as well as those with clinically inactive disease (CID). CD14+ cells were isolated by magnetic beads separations, and used to generate RNA which in turn was used to quantitate the expression of 384 miRNA and controls on the TaqMan ™ MicroRNA Array A (Life technologies).

## Results

We found several specific miRNA that have been implicated in monocyte/macrophage differentiation with increased expression in monocytes from children with active SJIA, including miR-27a, miR-125a-5p and miR 142-3p. We also found increased expression of several other miRNA that have been associated with pathogenesis of RA, including miR-223 which has been implicated in regulation of the NALP3 inflammasome, miR-26a, and miR-132. Interestingly, while monocyte expression of miR-146a has been correlated with disease activity in RA, we found no difference in expression between monocytes from patients with active SJIA and those with CID.

## Conclusion

These results provide the first report of miRNA expression profiles in children with SJIA. Taken together, these data suggest that differential miRNA expression contributes to the phenotype of monocyte/macrophages in SJIA, and may have implications for disease pathogenesis and development of MAS. Further work will correlate miRNA expression with clinical features and gene expression profiles, as well as examine impact of miRNA expression on monocyte function and differentiation.

## Disclosure of interest

G. Schulert: None declared., N. Fall: None declared., N. Shen: None declared., A. Grom Grant / Research Support from: Novartis, NovImmune, Consultant for: Novartis, Roche.

